# CART-Cell Therapy: Recent Advances and New Evidence in Multiple Myeloma

**DOI:** 10.3390/cancers13112639

**Published:** 2021-05-27

**Authors:** Massimo Martino, Filippo Antonio Canale, Caterina Alati, Iolanda Donatella Vincelli, Tiziana Moscato, Gaetana Porto, Barbara Loteta, Virginia Naso, Massimiliano Mazza, Fabio Nicolini, Andrea Ghelli Luserna di Rorà, Giorgia Simonetti, Sonia Ronconi, Michela Ceccolini, Gerardo Musuraca, Giovanni Martinelli, Claudio Cerchione

**Affiliations:** 1 Stem Cell Transplant and Cellular Therapies Unit, Hemato-Oncology and Radiotherapy Department, Grande OspedaleMetropolitano “Bianchi-Melacrino-Morelli”, 89124 Reggio Calabria, RC, Italy; filcan87@gmail.com (F.A.C.); tiziana.moscato@ospedalerc.it (T.M.); porto.tania25@gmail.com (G.P.); barbara.loteta@ospedalerc.it (B.L.); virginia.naso@ospedalerc.it (V.N.); 2 Hematology Unit, Hemato-Oncology and Radiotherapy Department, Grande Ospedale Metropolitano “Bianchi-Melacrino-Morelli”, 89124 Reggio Calabria, RC, Italy; caterina.alati@gmail.com (C.A.); donatella.vincelli@gmail.com (I.D.V.); 3 Immunotherapy, Cell Therapy and Biobank (ITCB), IRCCS Istituto Romagnolo per lo Studio dei Tumori (IRST) “Dino Amadori”, 47014 Meldola, FC, Italy; massimiliano.mazza@irst.emr.it (M.M.); fabio.nicolini@irst.emr.it (F.N.); 4 Biosciences Laboratory, IRCCS Istituto Romagnolo per lo Studio dei Tumori (IRST) “Dino Amadori”, 47014 Meldola, FC, Italy; andrea.ghellilusernadirora@irst.emr.it (A.G.L.d.R.); giorgia.simonetti@irst.emr.it (G.S.); 5 Hematology Unit, IRCCS Istituto Romagnolo per lo Studio dei Tumori (IRST) “Dino Amadori”, 47014 Meldola, FC, Italy; sonia.ronconi@irst.emr.it (S.R.); michela.ceccolini@irst.emr.it (M.C.); gerardo.musuraca@irst.emr.it (G.M.); giovanni.martinelli@irst.emr.it (G.M.)

**Keywords:** multiple myeloma, BCMA, CAR T, relapsed multiple myeloma, refractory myeloma, cytokine release syndrome, neurologic toxicity

## Abstract

**Simple Summary:**

Available data on anti-BCMA CART-cell therapy has demonstrated efficacy and manageable toxicity in heavily pretreated R/R MM patients. Despite this, the main issues remain to be addressed. First of all, a significant proportion of patients eventually relapse. The potential strategy to prevent relapse includes sequential or combined infusion with CAR T-cells against targets other than BCMA, CAR T-cells with novel dual-targeting vector design, and BCMA expression upregulation. Another dark side of CAR T therapy is safety. Cytokine release syndrome (CRS) and neurologic toxicity are well-described adverse effects. In MM trials, most CRS events tended to be grade 1 or 2. Another critical point is the extended timeline of the manufacturing process and that only a few academic centers can perform these procedures. Recognizing these issues, the excellent response with BCMA-targeted CAR T-cell therapy makes it a treatment strategy of great promise.

**Abstract:**

Despite the improvement in survival outcomes, multiple myeloma (MM) remains an incurable disease. Chimeric antigen receptor (CAR) T-cell therapy targeting B-cell maturation antigen (BCMA) represents a new strategy for the treatment of relapsed/refractory MM (R/R). In this paper, we describe several recent advances in the field of anti-BCMA CAR T-cell therapy and MM. Currently, available data on anti-BCMA CART-cell therapy has demonstrated efficacy and manageable toxicity in heavily pretreated R/R MM patients. Despite this, the main issues remain to be addressed. First of all, a significant proportion of patients eventually relapse. The potential strategy to prevent relapse includes sequential or combined infusion with CAR T-cells against targets other than BCMA, CAR T-cells with novel dual-targeting vector design, and BCMA expression upregulation. Another dark side of CART therapy is safety. Cytokine release syndrome (CRS) andneurologic toxicity are well-described adverse effects. In the MM trials, most CRS events tended to be grade 1 or 2, with fewer patients experiencing grade 3 or higher. Another critical point is the extended timeline of the manufacturing process. Allo-CARs offers the potential for scalable manufacturing for on-demand treatment with shorter waiting days. Another issue is undoubtedly going to be access to this therapy. Currently, only a few academic centers can perform these procedures. Recognizing these issues, the excellent response with BCMA-targeted CAR T-cell therapy makes it a treatment strategy of great promise.

## 1. Introduction

Several drugs have shown activity in multiple myeloma (MM) and are available for clinical use. As a result, there are numerous regimens that use two or more of these drugs available for the treatment of newly diagnosed and refractory/relapsed MM. The major classes include alkylating agents (melphalan andcyclophosphamide), corticosteroids (dexamethasone andprednisone), immunomodulatory drugs (thalidomide, lenalidomide, and pomalidomide), and proteasome inhibitors (bortezomib, carfilzomib, andixazomib) [1]. The treatment paradigm in MMis evolving with the introduction of various immunotherapies [2]. First came the monoclonal antibodies (mAbs), which represented a paradigm shift in treating all MM stages [3,4,5,6,7]. The initial mAbs included daratumumab, which targets CD38 on MM cells’ surface [8,9], and elotuzumab, which targets SLAMF7 [10]. These new molecules were followed by isatuximab, which also targets CD38 [11]. The new agents have clearly shown significant activity and improved progression-free survival (PFS) in newly diagnosed and relapsed MM.

Other immunotherapy drugs are poised to alter the landscape of MM treatment going forward significantly. Antibody-Drug Conjugates (ADCs) represent a new platform [12] and are best exemplified by belantamabmafodotin (BM) [13]. BM is a mAb targeting B-cell maturation antigen (BCMA) linked to a microtubule-disrupting agent, monomethyl auristatin F (MMAF) [14]. BCMA is a transmembrane glycoprotein and a member of the tumor necrosis factor (TNF) receptor superfamily, and it is preferentially expressed on mature B-cells and plasma cells in MM and plays an essential role in long-term plasma cell survival. The number of surface BCMA increases with the progression of the disease. Because of the localization to plasma cells and the expression on virtually no other cell type, BCMA can represent an ideal target for MM-specific cellular therapies [15]. BM as a singleagent has been associated with a response rate >30% in patients with multiple previous lines of therapy, including those who do not respond to PIs, IMiDs, and mAbs. The drug is now being studied in combination with other agents in both upfront and relapsed settings. Besides, several other conjugate drugs targeting BCMA are under investigation in clinical trials [16].

Another platform that has shown significant activity in patients with MM is bispecific antibodies (BiAbs) [17]. BiAbs are designed to bring T-cells closer to the tumor cell by targeting both cell types simultaneously, promoting a more robust T-cell response and immune synapse formation to eliminate MM cells. With BiAbs, investigators reported few CRS rates, easy management, and very little neurotoxicity.

**Figure 1:**
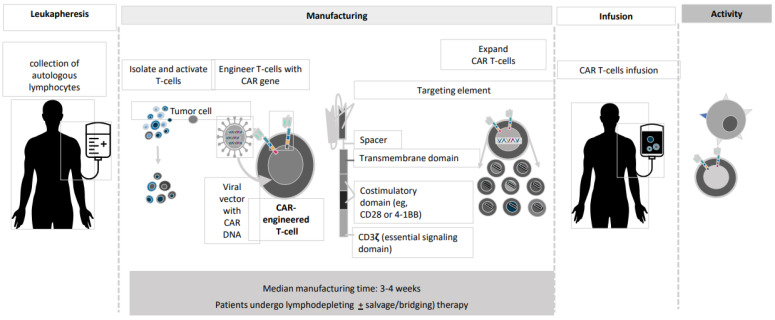
CAR T manufacturing process.

**Figure 2:**
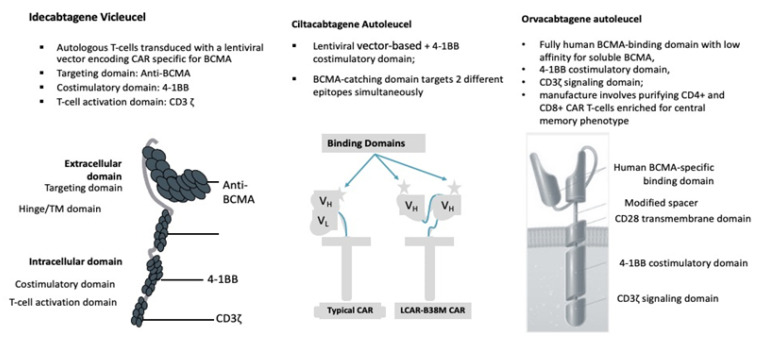
BCMA CAR T-cell Contruct Design.

And last but not least, CAR T-cells. CAR T-cell transplantation technology has evolved dramatically during the past 25 years (Figure 1) [18,19,20,21,22]. The current version of BCMA-directed CAR T-cell therapy employs a construct with a single-chain variable fragment that recognizes the BCMA antigen, a spacer, an intracellular co-stimulatory signaling domain (often 4-1BB), and a CD3ζ intracellular domain to stimulate T-cell activation upon binding (Figure 2). The initial wave of CAR T-cells that target BCMA has shown remarkable efficacy in MM. In this paper, we describe the several recent advances in CAR T-cell therapy and MM and provide a preview of some of the exciting research presented at recentinternational meetings.

## 2. Anti-BCMACAR T-Cell Studies

Outcomes for patients with triple-exposed relapsed or refractory (R/R) MM have been poor, with a median PFS of only 3 to 4 months [23]. Among R/R MM patients who have been exposed to IMIDs, PIs, mAbs, and have received multiple lines of therapy, there are no standard-of-care options. Often, the choice of treatment we offer is based on the availability of the utmost efficacious agent. Interest in CAR T-cell therapy in MM has been high, especially for products targeting BCMA. Several BCMA-targeted CAR T-cell therapies are in clinical development for patients with R/R MM, and trials’ results are published or presented at the most recent international congresses (Table 1 and Table 2).

### 2.1. Idecabtagene Vicleucel

The most extensive study is the KARMMA-2 trial with idecabtagenevicleucel (Ide-Cel), presented at the 2020 American Society of Clinical Oncology (ASCO) [24]. Ide-Cel (bb2121) induced responses in nearly three-fourths of patients with heavily pretreated, highly R/R MM, according to findings reported by Munshi and colleagues [24]. The global KarMMa trial enrolled 158 patients with at least three prior therapies, of whom 140 underwent leukapheresis, and 128 were treated at dose levels ranging from 150 to 450 × 10^6^ CAR-positive T-cells. The study population had received a median of six prior regimens; the majority had a high tumor burden, one-third had an extramedullary disease, and one-third had high-risk cytogenetics. Eighty-eight percent of patients required bridging therapy during the CAR T-cell manufacturing process. The study’s primary endpoint was OR, and the secondary endpoint was CR. With a median follow-up of 13.3 months across all dose levels, KarMMa met both endpoints, with an ORR of 73% (*p* < 0.0001) and a CR rate of 33% (*p* < 0.0001). Clinically meaningful efficacy was observed across subgroups. Median PFS increased with the higher CAR T dose. Median OS was 19.4 months, with 78% of patients alive at 12 months. Most patients developed some degree of Cytokine release syndrome (CRS), 78% of which were low grade. Neurologic toxicity(NT)developed in 18%, mostly low grade as well. Cytopenias grade ≥3 were expected, but the average patient recovered within three months. Infections were also common and were not thought to be dose-related. The results supported a favorable benefit-to-risk profile for Ide-Cell across the dose range of 150 to 450 million cells. In March 2020, the Investigators of Ide-Cel submitted a biologics license application to the U.S. Food and Drug Administration (FDA) to use Ide-Cel after at least three prior therapies, including an IMID, a PI, and a mAb. 

In the phase I CRB-402 study, patients with heavily pretreated relapsed/refractory MM had high rates of very good partial responses (VGPRs) or better to the anti-BCMA CAR T cell therapy bb21217, according to updated results presented at the 2019 American Society of Hematology (ASH) Annual Meeting [25]. At a follow-up ranging from 1 to 35 months, the tumor response was 68%, ranging from 43% to 83%, depending on the dose [26]. Higher doses appear to be associated with better responses. Patients with a higher proportion of memory-like T cells also had significantly better peak expansion. 

Although extending survival is the ultimate treatment goal of MM therapy, reducing disease-related symptoms and improving quality-of-life (QoL) are very important. MM patients have a high symptom burden, associated with reduced overall health-related QoL (HRQoL), particularly concerning physical functioning [32]. Shah et al. [33] reported the impact of Ide-Cel treatment on secondary HRQoL domains of interest and health utility scores in patients with R/R MM in the KarMMa trial, and the results showed that Ide-Cel treatment brings clinically meaningful QoL benefits to triple-class-exposed patients with R/R MM without compromising any HRQoL domains.

### 2.2. Ciltacabtagene Autoleucel

Ciltacabtageneautoleucel (JNJ-4528) (Cilta-Cel) is an investigational CAR T-cell therapy comprising a 4-1BB co-stimulatory domain and two proteins that attach to BCMA to confer avidity. It is identical to the CAR T-cell construct LCAR-B38M, which also showed activity in the Chinese LEGEND-2 study, reported at the 2019 American Society of Hematology (ASH) Annual Meeting [34]. CARTITUDE-1 was a phase Ib/II trial conducted in the United States [27,35]. The study enrolled 113 patients with R/R MM with measurable disease, ECOG PS ≤1; ≥3 prior therapies including PI, IMiD, a mAb therapy, or double refractory to PI and IMiD. After the screening, enrollment, patients underwent leukapheresis. During Cilta-Cel manufacturing, 73 patients received bridging therapy with previously used agents to maintain a stable disease. Of 113 patients enrolled, 97 received Cilta-Cel (phase Ib, *n* = 29; phase II, *n* = 68) with a median administered dose of 0.71 × 10^6^ (0.51–0.95 × 10^6^) CAR+ viable T-cells/kg. The primary endpoints were safety and recommended phase II dose (RP2D) in phase Ib and efficacy in phase II. At the 2020 ASCO Annual meeting, Berdeja et al. [27] presented the results for 29 patients whit a longer follow-up than the first report at the 2019 ASH Annual Meeting. Patients had received a median of five prior therapy lines (range, 3–18 lines). As a prior treatment, 86% had undergone an autologous (Auto)stem cell transplantation (SCT); 100% were triple-exposed, and 86% were refractory to three standard treatments; 76% were penta exposed, and 31% were refractory to five agents. After lymphodepletion, the patients received a single infusion of the JNJ-4528 CAR T-cells at a targeted dose of 0.75 × 10^6^ CAR-positive cells/kg. The ORR was 100%, with sCR achieved by 86% and at least a VGPR in 97%. Response rate and depth of response were independent of BCMA expression at baseline. In the initial analysis reported at the 2019 ASH Annual Meeting [28], 100% of patients were MRD-negative, and the majority continue to show an MRD-negative response by day 28. Based on initial findings from CARTITUDE-1, JNJ-4528 received breakthrough therapy designation by the FDA to treat patients with R/R MM. Madduri et al. presented the CARTITUDE-1 study update at the 2020 ASH Annual Meeting [28]. Investigators concluded that Cilta-Cel confirmed a manageable safety profile at RP2D (0.75 × 10^6^ viable CAR T-cells/kg). Most cases of CRS were grade 1/2 with a median time to onset of 7 days. Twenty (20.6%) patients experienced CAR T-related NT, and 10.3% had grade ≥3 events. In heavily pretreated patients with R/R MM, responses were early, deep, and durable. The ORR was 96.9% with ≥ a 67% sCR rate. Median PFS was not reached; 12-mo PFS and OS rates were 76.6% and 88.5%, respectively. Studies are ongoing to evaluate Cilta-Celin earlier-line settings for other populations of patients with MM, and administration of Cilta-Celin outpatient setting is being assessed in ongoing phase II/III studies (NCT04133636; NCT04181827).

### 2.3. Orvacabtagene Autoleucel

OrvacabtageneAutoleucel (Orva-Cel) is a BCMA-directed CAR T-cell product with a fully human binder. The construct reduces antigen-independent exhaustion by minimizing tonic signaling and improving binding to BCMA on target cells. A novel manufacturing process has allowed for a purified CD4+ and CD8+ CAR T-cell product enriched fora central memory phenotype, good persistence, and durability. EVOLVE (NCT03430011) is a phase I/II study investigating different dose ranges of CAR T-cells in patients with R/RMM. The previously published results for lower doses have demonstrated an acceptable safety profile with promising clinical activity [29]. Mailankody and colleagues presentedtheir results from patients treated with higher doses atthe 2020 ASCO Annual Meeting [30]. Orva-Cel therapy was infused at three different doses, manufactured using the same process intended for commercial use. A total of 62 patients were included in the study. The most common adverse events were hematologic toxicities and CRS (any grade), with similar frequency across three dose groups. The objective response rate was 92% for all dose groups, and 68% of patients reached at least a VGPR. Of all evaluable patients, 84% were measurable MRD negative at three months post-infusion. Persistent CAR T-cells were detected in 69% of evaluable patients six months after infusion. All patients with high baseline soluble BCMA responded to the treatment.

### 2.4. Results from the Last International Meeting and Novel BCMA-Targeted CAR T-Cell Product

During the 2020 ASH annual meeting, an impressive number of presentations detailing clinical research into this therapeutic modality havebeen presented.

Lin et al. [31] reported updated safety and efficacy results for 62 patients who received Ide-Cel in the ongoing CRB-401 study. Among all 62 patients, the ORR was 76%. The median duration of response was 10.3 months. Of 37 responders evaluable for MRD, 30 were MRD negative at one or more time points, and seven responders were MRD positive. With a median follow-up of 14.7 months for all patients in the dose-escalation and dose-expansion phases, median PFS was 8.8 months, and median OS was 34.2 months. Overall, a dose-dependent effect was observed on responses and survival outcomes, with greater efficacy and a favorable clinical benefit-risk profile for Ide-Cel at target dose levels ≥ 150 × 10^6^ CAR T-cells.

bb21217 is a BCMA-targeted CAR T-cell product with the same CAR molecule as Ide-Cel (bb2121) that has been cultured with PI3K inhibitor to enrich for T-cells with memory-like phenotype. The construct is a CAR with a memory-like phenotype associated with persistence after infusion and sustained response and remission in patients with CLL treated with CAR T-cell therapy [36]. CRB-402 was the first-in-human phase I trial evaluating bb21217 in heavily pretreated patients with R/R MM [25,37]. bb21217 associated with high efficacy rate, CAR T-cell persistence in patients with long-term response, and manageable safety profile. At the ASH 2020 Annual Meeting, investigators presented the current analysis of the ongoing CRB-402 trial with updated safety and efficacy data and biomarkers for T-cell expansion and durable response with bb21217 [38]. In detail, CRB-402 was a multicenter, open-label, first-in-human, dose-escalation/-expansion phase I trial. The trial enrolled patients with R/R MM with three or more prior regimens, including IMiD and PI; ≥50% BCMA expression (dose-escalation only); prior anti-mAb and refractory to last line of treatment (dose-expansion only) (*n* = 69 infused by cutoff). Primary endpoints were the rate of AEs, dose-limiting toxicity. Most common AEs included neutropenia, pyrexia, CRS, thrombocytopenia, anemia, and leukopenia. Cytopenias were observed in a range of 35% to 80% of patients, independent of dose. Investigators observed response rate higher, responses more profound, and similar safety profile in patients treated after versus before manufacturing change. Patients experiencing response had significantly greater peak vector copy numbers than those without response (*p* < 0.0001). Patients receiving 450 × 10^6^ cells achieved higher sustained vector copy numbers vs. those receiving 150 or 300 × 10^6^ cells. In patients with a more significant proportion of CAR T-cells with a memory-like phenotype, peak expansion significantly higher (both *p* ≤ 0.0024 for a percentage of CD4+ and CD8+ central memory T-cells). Patients with sustained response had T-cells exhibiting a memory-like phenotype as determined by naive vs. effector gene signature. In this updated analysis of the ongoing CRB-402 trial, the safety profile for bb21217 was consistent with known CAR T-cell–associated toxicities. High response rate and in-depth responses were observed at RP2D of 450 × 10^6^, with ORR at RP2D after manufacturing process change of 84%. Patients with a more significant proportion of T-cells exhibiting memory-like phenotype exhibited greater CAR T-cell expansion and durable response. The authors’ conclusions supported the hypothesis that was enriching T-cells with memory-like phenotypes, like with bb21217 CAR T-cell product, leads to improved CAR T-cell persistence, expansion, and durability of response. The enrollment has been completed, and follow-up ongoing for the CRB-402 trial.

Lam et al. [39] used the sequence of a novel anti-BCMA, fully-human, heavy-chain-only binding domain designated FHVH33. FHVH33 lacks the light chain, artificial linker sequence, and two associated junctions of an scFv. The objective was to reduce the risk of recipient immune responses against CAR T-cells and be less immunogenic than an scFv. Investigators constructed a CAR incorporating FHVH33, CD8α hinge and transmembrane domains, a 4-1BB co-stimulatory domain, and a CD3ζ T-cell activation domain. The CAR, FHVH33-CD8BBZ, was encoded by a γ-retroviral vector. FHVH33-CD8BBZ-expressing T-cells (FHVH-BCMA-T) exhibited a full range of T-cell functions in vitro and eliminated tumors and disseminated malignancy in mice. Mikkilineni et al. designed the first clinical dose-escalation trial of FHVH-BCMA-T [40]. The median age of patients enrolled was 63 (range 52–70); patients received a median of six anti-myeloma therapy lines (range 3–10) before treatment with FHVH-BCMA-T. ORR was observed in ten out of twelve patients. Five patients have obtained CRs or VGPRs to date. A response also occurred in patients with large soft-tissue plasmacytomas. Eleven out of 12 patients had CRS, and one patient had grade 3 neurotoxicity. Toxicity was limited and reversible. The results from this phase 1 trial demonstrated that FHVH-BCMA-T-cells could induce responses at low dose levels. Accrual to this trial continues, and a maximum tolerated dose has not been determined yet. These results encourage further development of FHVH CAR T.

CT053 comprises autologous T-cells genetically modified with a second-generation CAR incorporating a fully human BCMA-specific single-chain fragment variant (25C2) with high binding affinity. Twenty-four subjects have been treated in phase 1 studies with an 87.5% ORR, 79.2% CR, and a median duration of response of 21.8 months without inducing immunogenicity [41]. Kumar et al. presented the results of the ongoing phase 1b/2 study (LUMMICAR-2) conducted in North America (NCT03915184) to evaluate the safety and clinical efficacy [42]. At the data cutoff, ten subjects were evaluable for at least two months of efficacy assessment with a median follow-up of 4.5 months (range 2–8). A 100% ORR was observed, with two sCR, 1 VGPR, and 5 PR. Of the 12 subjects with evaluable samples, 11 were MRD-negative at the 10^5^ sensitivity level. Responses were independent of baseline bone marrow BCMA expression. CT053 transgene levels showed the peak expansion at 7–14 days after infusion. All subjects showed similar kinetics of rapid declines in serum-free light chains and soluble BCMA levels within one month, and continued depletion in sBCMA suggests CT053-mediated pharmacodynamic activity.

### 2.5. Novel Anti-BCMACAR T Therapies

CAR T-cell therapies comprised of a high percentage of stem memory T-cells (T_SCM_). T_SCM_ appear to provide more excellent response rates, more extended response duration, and increased safety [43,44,45,46]. P-BCMA-101 is a novel anti-BCMA CAR T-cell therapy manufactured using transposons instead of viral vectors, intended to transpose T_SCM_ cells preferentially, include a safety switch to turn off CAR T-cells, and a drug resistance gene to allow for positive selection of CAR T-cells during manufacturing. The manufacturing process changed using nanoplasmid to improve transposition efficiency. PRIME was an I/II Trialdesigned to evaluate the safety and efficacy of escalating doses of P-BCMA-101 in patients with R/R MM [47]. The cohort study consisted of patients with R/R MM, three or more prior therapy lines (including PI+IMiD), or two or more prior therapy lines in patients refractory to both PI+IMiD, ECOG PS 0−1. P-BCMA-101 was initially given at escalating single-infusion doses 0.75−15 × 10^6^ cells/kg and subsequently at a median dose of 0.75 × 10^6^ cells/kg in biweekly cycles. Percentage of T_SCM_ in CAR T-cell dose correlated with the probability of response. Investigators concluded that in the phase I/II PRIME trial, P-BCMA-101 was safe and effective. The CRS rate was 17%, all grade 1/2, managed with tocilizumab/steroids without need for ICU admission. The use of T_SCM_ could increase the response rate and safety of CAR T-cell therapy, and CAR T-cell expansion and efficacy could increase with a change to the manufacturing process using nanoplasmids. ORR was 67%, with VGPR or sCR 50%, versus 50% (all PR) with standard plasmid. The dosing strategy did not affect safety outcomes, and multiple doses did not appear to affect the pharmacokinetics of efficacy. Dose escalation is ongoing in patients receiving nanoplasmids.

## 3. The Future

The most clinic advanced trials in MM are using the anti-BCMA CAR T-cell products bb2121 [48] and JNJ-4528 [49] (Table 3).

However, these agents, amongst others, are also being explored in early lines of therapy [50,51,52]. One way to maximize the efficacy of CAR T-cell therapy may be to combine CAR T-cells with other agents [53,54], such as a gamma-secretase inhibitor (GSI) [55]. The use of GSI might increase expression of BCMA on the myeloma cell surface while also decreasing the levels of sBCMA in circulation, overall leading to increased efficacy of anti-BCMA CAR T. Novel therapies targeting molecules beyond CD19 and BCMA, including CD4 [56], CD38 [57], CD44v6 [58], CD138 [59], SLAMF7 [60], and others are under investigation (Table 4).

Additional strategies in clinical development include CAR T-cell cocktails, as well as dual and bispecific CARs. Results from some dual targeting approaches have already been presented at recent meetings, such as the SZ-CART-MM02 study (NCT03455972), presented during the forty-fifthmeeting of the European Society for Blood and Marrow Transplantation (EBMT) [61]. The treatment regimen included induction therapy and Auto-SCT, followed by anti-CD19 and anti-BCMA CAR T-cells in patients with “de-novo” MM. The ORR was 100% (10/10 patients). MRD negativity rate was 44.4% after Auto-SCT and 60.0% after CAR T infusion. CRS occurred in 100% of patients (10/10), with no cases of CRS ≥ 3. It was shown that the CAR T-cells were amplified and could be detected for more than one year after infusion. In patients with high-risk disease, CAR T-cells’ peaks appeared later but were sustained for longer than patients with R/R disease. This study showed that tandem Auto-SCT with anti-CD19 and anti-BCMA CAR T-cell infusion might provide an alternative consolidation treatment for high-risk MM patients. The authors hypothesize that the immune system may be remodeled following Auto-SCT, contributing to a higher CAR T-cell expansion.

Jiang and colleagues have designed a dual FasTCAR T targeting both BCMA and CD19 [62]. The BCMA-CD19 dual CAR was constructed by linking BCMA and CD19 scFv, joined by a CD8 hinge, transmembrane domain, co-stimulatory domain, and CD3z. At the 2020 ASH annual meeting, investigators reported early results from the first-in-human multicenter clinical study (NCT04236011; NCT04182581) BCMA-CD19-directed FasTCAR T (GC012F) in 16 patients with R/R MM. CART-cells were administered in a single infusion at three dose levels. The BCMA-CD19 dual FasTCAR T data showed an early and high response rate with 93.8% ORR to date with a promising early high MRD-sCR rate in the highest dose level (100%), which was sustained with a median duration of follow up of 7.3 months at cut off. The data shows an auspicious activity of the BCMA-CD19 dual FasT CART with a favorable safety profile, and 93.8% (15/16) of the treated patients exhibited high-risk features. This data indicated that BCMA-CD19 dual FasTCAR T (GC012F) might present an effective new treatment option for patients with R/R MM, including those with high-risk features that failed multiple prior therapies. The study is still ongoing and enrolling patients.

Although anti-BCMA CAR T-cell therapy has proven efficacious, access is limited by logistics, wait time, and bridging treatment. Allogeneic (Allo) CAR T-cell therapies are generated from healthy donor T-cells. This approach is hypothesized to overcome some of the challenges experienced with traditional CAR T therapy, such as the potentially long time between apheresis and product manufacturing.The UNIVERSAL study was the first-in-Human Phase I Trial of Allo-CAR T-Cell Therapy with ALLO-715 (Anti-BCMA) and ALLO-647 (Anti-CD52) in patients with R/R MM [63]. Allo-CAR T-cells were well tolerated and showed promising activity in heavily pretreated patients. The trial was a multicenter, open-label, dose-escalation phase I study that enrolled adults with three or more previous therapies (including IMiD, PI, and mAb) and refractory to last therapy. Twenty-six patients reached the assessment point and were included in the efficacy analysis. Lymphodepletion consisted of Flu (30 mg/m^2^ × 3 days) + Cy (300 mg/m^2^ × 3 days) or Cy regimen alone + ALLO-647 13−30 mg × 3 days. Treatment consisted of a single ALLO-715 Infusionon Day 0, 40, 160, 320, 480 × 10^6^ CAR T-cells. The primary endpoint was safety and tolerability. Secondary endpoints were lymphodepletion regimen and recommended ALLO-715 phase II dose; anti-tumor activity (ORR, DoR, PFS, and MRD); ALLO-715 cellular kinetics; and ALLO-647 PK data. In this study, 90% of patients received CAR T-cell infusion within five days of enrollment. No GVHD or ICANS occurred; 43% experienced grade 1/2 CRS, considered manageable with low use of tocilizumab (19%) and steroids (10%). Ninteen percent of patients had serious AEs (grade ≥ 3); in five of them (16%) had grade ≥3 infection, and one had grade 5 events related to progressive myeloma. ALLO-715 activity increased with greater doses; 60% of patients given 320 × 10^6^ dose after Flu + Cy lymphodepletion achieved response; fiveof six patients achieving ≥VGPR were MRD negative. Cell expansion occurred on day 7 after dosing and persisted for more than four months. Investigators concluded “off-the-shelf” anti-BCMA CAR T-cell therapy feasible in MM, and further studies to assess higher celldoses and lymphodepletion are currently enrolling patients.

NK cells are being investigated as an alternative to traditional T-cell approaches to overcome CAR T therapy’s adverse events, such as cytokine release syndrome [64,65,66]. There is currently one trial in China investigating CAR NK cells for patients with MM listed as ‘recruiting’ on the clinical trials database. NCT03940833 is a phase II study of anti-BCMA CAR-NK 92 cells in patients with RR/MM [67].

## 4. Expert Opinion

In the last few years, the incorporation of several therapies, including IMIDs, PIs, and mAbs, has improved the outcomes of MM. Nevertheless, patients who become refractory to new agents are increasingly less responsive to subsequent therapies. There is an urgent need for new approaches in this setting, and available data on anti-BCMA CART-cell therapy have demonstrated efficacy and manageable toxicity in heavily pretreated R/R MM patients. The CAR T field in MM is rapidly evolving with an impressive number of ongoing clinical trials across all phases, different CAR T designs, or targets. State of the art, anti-BCMA CAR-T-cell studies show an unprecedented efficacy, with a response greater than 90%.

Despite this, the main issues remain to be addressed (Table 5). First of all, durability has remained an ongoing clinical dilemma because a significant proportion of patients eventually relapse, and we do not know wherein the sequence of MM therapy CAR T-cell should fall. Although all of the studies showed high initial CR and MRD negative status, relapses occur at a relatively high rate. CAR T-cell therapy failure is multifactorial, involving malignancy, immune-associated, and patient-factors. Antigen escape is one underlying mechanism of relapse after cellular immunotherapy. Downregulation or loss of BCMA has been observed in patients who relapsed after CAR T [68,69,70,71]. We believe that lack of CAR T persistence is an important contributing factor to relapse, and the immunosuppressive effects of the tumor microenvironment and malignant plasma cells on CAR T-cells’ function play a role in the resistance to immune-based therapy in patients with MM [18].

The strategies to overcome an antigenic loss in relapse after CAR T-cell therapy include: (1) sequential or combined infusion with CAR T-cells against targets other than BCMA; (2) CAR T-cells with novel dual-targeting vector design; (3) and BCMA expression upregulation. Several antigens (CD138, GPRC5D, transmembrane activator, calcium-modulator, cyclophilin ligand, signaling lymphocytic activation, and molecule) NKG2D ligands, CD229, integrin β have been identified and explored as potential targets of immunotherapy. Some of them, such as GPRC5D, is expressed on the surface of CD138+ multiple myeloma cells, independent of BCMA expression, but it is minimally expressed in other cell lines [72]. Dual-targeted T-cells are actively being investigated, and recently, some groups reported preclinical data investigating dual-targeting approaches for CAR T-cell therapy, using BCMA and GPRC5D as a model [73]. Although many patients respond to CARs, some have not long-lasting responses, and many relapses despite achieving in-depth responses. We may overcome both of these problems by moving CAR T-cell therapy into the earlier treatment lines. Indeed, ongoing phase III trials are evaluating CAR T-cell therapy in patients with two or more previous lines of therapy, as well as in newly diagnosed high-risk patients.

Another dark side of CAR T therapy is safety. CRS and NT are well-described adverse effects of CAR T-cell therapy, regardless of target or tumor type. In the MM trials, most CRS events tended to be grade 1 or 2, with fewer patients experiencing grade 3 or higher. Most patients with CRS were easily managed with tocilizumab and other standard measures. NT has not been a significant issue in any of these MM CAR T-cell trials, with most cases responding to current management measures. It is also clear that with experience, the management of adverse events such as CRS and NT has become less burdensome, and these procedures are now much safer for patients. Although toxicities occur frequently, these adverse events appear very manageable, and severe non-hematologic toxicity is increasingly rare. Fromthese results, many centers postulate the possibility that CAR T therapies are managed on an outpatient basis orwith a reduction in the days of hospitalization.

However, it is important not to forget that there are long-distance ones, such as cytopenias and hypogammaglobulinemias, in addition to short-side effects. These clinical conditions must be monitored carefully and lead to a worse quality of life for our patients.

Another critical point is the extended timeline of the manufacturing process. This treatment waiting-time can be challenging for patients with rapidly progressing R/R disease who need immediate treatment. There is also an attempt to avoid the need for apheresis and the extended manufacturing process by using allogenic CAR T-cells. Manufacturing CAR T-cells using lymphocytes from allogeneic (Allo) donors have long been investigated in various malignancy types, including MM. The T-cells needed for Allo-CAR T are harvested from healthy donors and genetically engineered to express CARs aimed at specific cancer targets [74]. Several novel bioengineering methods (i.e., knocking out the T-cell receptor and major histocompatibility complex expression using various gene-editing techniques) have been implemented to moderate potential graft-versus-host toxicity and host rejection. The off-the-shelf CART-cell ALLO-715, which targets BCMA, demonstrated responses as treatment of patients with heavily pretreated R/R MM in a first-in-human clinical trial, according to the early findings presented at the 2020 ASH Annual Meeting. The findings mark the first results for an Allo-CAR therapy directed at BCMA that has sparked intensive research interest. The advantage of the ALLO-CAR T can be summarized as follows: (1) access (potential to treat all eligible patients; repeat dosing, if needed; no need for complex logistics); (2) cost (scalable and efficient manufacturing; potential to treat 100+ patients from a single manufacturing run; lower ancillary costs); (3) speed reliability (off-the-shelf for on-demand treatment; less product variability, made from healthy T-cells); (4) innovation (multiplex gene-engineering and gene-editing capabilities; opportunity for product optimization).

CRISPR is a highly precise gene-editing tool that is changing cancer research and treatment and could be in the future an alternative to CAR T therapy [75]. With CRISPR, we can generate off-the-shelf (allogeneic) CAR-T-cells, which have distinct advantages over the autologous (patient-derived) products currently on the market. In addition, we can use CRISPR to eliminate or insert genes to create new classes of CAR T products with improved applicability to several tumors. A CAR has two key domains: one that binds to the surface of cancer cells and another that activates the T-cell. The current generation of CAR T products uses randomlyintegrating viruses to deliver the CAR construct to the DNA of T-cells. In contrast, we can use CRISPR to insert the CAR construct precisely into the TCR alpha constant locus, which we expect to result in a safer, more consistent product. Donor T-cells could also recognize a patient’s cells as foreign through this receptor, leading to an unwanted side effect known as graft versus host disease (GvHD). We use CRISPR/Cas9 to eliminate the TCR with high efficiency, which reduces the risk of GvHD occurring during off-the-shelf use. MHC I: To improve CART-cell persistence and increase the chance for durable remissions, we could use CRISPR/Cas9 to eliminate the class I major histocompatibility complex (MHC I) expressed on the surface of our CAR T product candidates. If present, MHC I could lead to rejection of the CAR T product by the patient’s own T-cells. Eliminating this molecule should mitigate that effect. With all of its advantages over other gene-editing tools, CRISPR has become a go-to for scientists studying cancer. There is also hope that it will have a place in treating cancer.

In addition to donor-derived immune effector cells, induced pluripotent stem cell (iPSC)-derived immune cells are a promising platform for adoptive cellular therapy [76]. iPSC-derived lymphocytes have three critical advantages: (1) “off the shelf” availability; (2) a unique via clonal selection with a highly selected, multiply gene-edited, and consistent tumor-specific immune cell product; (3) and potent anti-tumor activity similar to conventional CAR T-cells, with maintaining of the innate phenotype, which can translate into fewer concerns about GVHD [77,78]. Another scenario could be represented by CAR NK cells, with a potentially lower CRS risk than conventional CAR T-cells. Several preclinical studies demonstrated cytotoxic activity and myeloma cell growth inhibition using CAR NK cells against various targets [65,66].

Another issue is undoubtedly going to be access to this therapy. Only a few academic centers can currently perform these procedures, although patients with R/R MM continue to grow. Recognizing these challenges, the excellent response with BCMA-targeted CAR T-cell therapy makes it a treatment strategy of great promise.

CAR T-cells represent an exciting single infusion (“one and done”) personalized therapy, with a potentially persistent, targeted immune-cytotoxicity. The disadvantages are the fast accredited center required (hospitalization likely required), CRS and NT with requires ICU and Neurology services; dependent on T-cell health (manufacturing failures), the need of significant support social (caregiver required), and last but not least, high cost. What more do we need? Firstly, improved CARs with faster manufacturing or off-the-shelf, better T-cells, persistence (a good “second wave”). Secondly, we need to improve the patient selection with a lower disease burden to prevent early relapse. It is important to investigate the role in the frontline setting and answer whether CARs can replace Auto-SCT. Third, we need trials with additional targets and combinations, such as GPRC5D ± BCMA, CD19 + BCMA, CAR + BISPECIFIC, and CAR + CelMOD. Finally, integration of CART with other myeloma therapies is an important area of future research [79]. Moreover, biomarkers for new immunotherapies could predict their efficacy in MM and define patients who would benefit from them. Several observations support the need for accurate immune profiling at baseline to try to identify ideal candidates and immune monitoring to identify those who would benefit the most from these agents [80].

## 5. Conclusions

Several studies confirm the efficacy of BCMA-CAR T-cell therapy in R/R MM. Activity was observed across high-risk subgroups, including high tumor burden, high-risk cytogenetics, and extramedullary disease. Longer follow-up is needed to assess long-term responses. Remission-inducing therapy is an unmet need for patients with myeloma that is refractory to proteasome inhibitors, immunomodulatory agents, and an anti-CD38 monoclonal antibody, as well as to prior autologous stem cell transplantation. CAR T-cell products targeting myeloma offer considerable promise for these patiernts.

## Figures and Tables

**Table 1:** Anti-BCMACAR T-Cell Studies: Main Patients’ characteristics.

	Idecabtagene Vicleucel (bb2121)						Ciltacabtagene Autoleucel (JNJ-4528)	Orvacabtagene Autoleucel (JCAR-H125)			Idecabtagene Vicleucel (ide-cel, bb2121)
**Author (year)**	Munshi (2020)			Berdeja (2019) Alsina (2020)			Berdeja (2020) Madduri (2020)	Mailankody (2020)			Lin (2020)
**Reference**	[24]			[25,26]			[27,28]	[29,30]			[31]
**Study Name**	KarMMa			CRB-402			CARTITUDE-1	EVOLVE			CRB-401
**Construct**	The ide-cel CAR is comprised of a murine extracellular single-chain variable fragment (scFv) specific for recognizing BCMA, attached to a human CD8 α hinge and transmembrane domain fused to the T-cell cytoplasmic signaling domains of CD137 4-1BB and CD3-ζ chain, in tandem			Cells engineered with bb2121 construct are then ex vivo cultured with PI3K inhibitor bb007			2 BCMA-targeting single-domain antibodies to boost avidity plus a 4-1BB co-stimulatory domain	Comprising fully human BCMA-binding domain with low affinity for soluble BCMA, 4-1BB co-stimulatory domain, CD3ζ signaling domain			The same characteristics asthe KarMMa study
**Median Age**	61 (range 33–78)			62 (range, 33–74)			61 (range, 43–78)	61 (range, 33–77)			61
**Cell Dose × 10^6^ kg**	150	300	450	150	300	450	0.75	300	450	600	50, 150, 450, or 800 × 10^6^ in the dose-escalation phase 150 to 450 × 10^6^ in the dose-expansion phase.
**No. Patients**	4	70	55	12	28	20	97 (29 phase Ib/68 phase II)	19	18	7	21 patients dose-escalation phase; 41 dose-expansion phase.
**Lymphodepletion**	FLU + CY			FLU + CY			FLU + CY	FLU + CY			FLU + CY
**Median Followup**	13.3			8.5			11.5	9.5	8.8	2.3	14.7

B-cell maturation antigen (BCMA); Lymhodepletion consisted of Fludarabine (FLU) 30 mg/m^2^ × 3 days + Cyclophosphamide (CY) 300 mg/m^2^ × 3 days

**Table 2:** anti-BCMA CAR T-Cell Studies: outcomes.

Car t Cell Construct	Idecabtagene Vicleucel (bb2121)						Ciltacabtagene Autoleucel (JNJ-4528)	Orvacabtagene Autoleucel (JCAR-H125)			Idecabtagene Vicleucel (ide-cel, bb2121)
**Author (Year)**	Munshi (2020)			Berdeja (2019) Alsina (2020)			Berdeja (2020) Madduri (2020)	Mailankody (2020)			Lin (2020)
**Study Name**	KarMMa			CRB-402			CARTITUDE-1	EVOLVE			CRB-401
**Reference**	[24]			[25,26]			[27,28]	[29,30]			[31]
**Response Rate**											
ORR	50%	69%	82%	83%	43%	73%	97%	95%	89%	92%	76(%)
CR	25%	29%	39%	18%			67%	37%	42%	29%	39(%)
Median DoR	NR	9.9	11.3	11.9			NR	NR	NR	NR	10.3(%)
Median PFS	2.8	5.8	12.1	NR	NR	NR	NR	9.3	NR	NR	8.8 (%)
Evaluable for MRD	4	70	54	7	6	4	57	11	11	3	37
MRD-	50%	31%	48%	100%	83.3%	100%	93%	72.7%	90.9%	100%	81%
**CRS Event**											
All	50%	76%	96%	67%			94.8%	89%			76(%)
Median Time to First Onset	7 (2–12)	2 (1–12)	1 (1–10)	3 (1–20)			7 (1–12)	2 (1–4)			Nk
Grade 3–4	0	4%	6%	2%			4%	3%			6(%)
Grade 5	0	1%	0	2%			1%	0			0
**Neurotoxicities**											
All	0	17%	20%	22%			20.6%	13%			44%
Median Time to First Onset	NA	3 (1–10)	2 (1–5)	7 (3–24)			8 (3–12)	4 (1–6)			Nk
Grade 3–4	0	7%	12%	4%			10.3%	3%			3%
Grade 5	0	0	0	2%			0	0			0

B-cell maturation antigen (BCMA); median time to first onset expressed in days (range); median age expressed in years (range); median follow up and progression-free survival (PFS) expressed in months; overall response rate (ORR); complete remission (CR): median duration of response (DoR) expressed in months; not known (Nk).

**Table 3:** anti-BCMA CAR T-cell therapy in multiple myeloma: ongoing trials.

CAR T Product	NCT Reference	Trial Name	Phase	Patient Population	Study’s Design/Primary Outcome
bb2121	NCT03651128	KarMMa-3	III	RRMM who had received 2–4 prior regimens, including ≥ 2 consecutive cycles of daratumumab, an immunomodulatory agent, and a Proteosm inhibitor, individually or in combinations	Arm A: bb2121 (est. enrolment: 254) Arm B: SOC therapy-DPd, DVd or IRd (est. enrolment: 127)
JNJ-4528	NCT04181827	CARTITUDE-4	III	RR/MM who have received 1 to 3 prior lines of therapy, including a proteasome inhibitor, and an immunomodulatory drug	Arm A: SOC therapy PVd or DPd Arm B: JNJ-4528 Primary outcome: PFS
bb21218 With lenalidomide maintenance	NCT04196491	KarMMa-4	I	High-risk ND/MM defined as R-ISS stage III per IMWG criteria	Rate of DLTs
JNJ-4528 Cohort D will also receive lenalidomide maintenance	NCT04133636	CARTITUDE-2	II	Cohort A: PD after 1–3 lines of therapy Cohort B: Early relapse after frontline therapy Cohort C: RR/MM after PI, IMiD, dara, and anti-BCMA therapy Cohort D: ND/MM after ASCT frontline therapy	Percentage of patients with negative MRD status
Anti-BCMA CAR T ± huCART19	NCT03549442	Not listed	I	Patients responding to first- or second-line therapy for high-risk MM	Number of AEs
Anti-BCMA CAR T Additional agent: Immune inhibitors	NCT03943472	Not listed	I	RRMM following ≥3 prior therapies including alkylating agents, PIs, and IMiDs with disease progression in the past 60 days	Safety (by number of AEs)
EGFRt/BCMA-41BBz CAR T-cell Additional agent:Lenalidomide	NCT03070327	Not listed	I	RRMM following >2 prior lines of treatment including IMiD and PI with refractory, persistent, or progressive disease	Determine the MTD of CAR T-cells
BCMA-specific CAR-expressing T Lymphocytes Additional agent: Gamma secretase inhibitor (JSMD194)	NCT03502577	Not listed	I	RR/MM following ASCT or transplant, ineligible patients with persistent disease after 4 cycles of induction that are refractory to both PI and IMiD therapy	Determine the MTD

DPd: daratumumab, pomalidomide, and low-dose dexamethasone; DVd: daratumumab, bortezomib, and low-dose dexamethasone; est.: estimated; IRd: ixazomib, lenalidomide, and low-dose dexamethasone; PFS: progression-free survival; PVd: pomalidomide, bortezomib, and low-dose dexamethasone; SOC: standard of care; R/R MM: relapsed/refractory multiple myeloma; ASCT: autologous stem cell transplant; AE: adverse event; BCMA: B-cell maturation antigen; CAR: chimeric antigen receptor; CR: complete response;dara: daratumumab; DLT: dose-limiting toxicity;IMiD: immunomodulatory drug; IMWG: International Myeloma Working Group; MM: multiple myeloma; MRD: measurable residual disease; NDMM: newly diagnosed multiple myeloma; PD: progressive disease; PI: proteasome inhibitor; R-ISS: revised International Staging System; RRMM: relapsed/refractory multiple myeloma.

**Table 4:** Single-targeting, non-anti-BCMA CAR T-cell therapy in patients with RRMM.

CAR T Product	NCT Reference	Antigen Target	Phase	Patient Population	Primary Outcome
CAR2 anti-CD38 A2 CAR T Cells	NCT03464916	CD38	I	RR/MM (following prior lenalidomide, pomalidomide, bortezomib, carfilzomib, and daratumumab) or RR/MM (within 1 year of high-dosefirst-line or second-line therapy/ASCT)	Determine the MTD
MLM-CAR44.1 T-cells	NCT04097301	CD44v6	I/II	RR/MM (≥4 different prior treatments in 3 treatment lines, or 4 treatments in 2 treatment lines in early relapsing patients)	MTD and recommended phase IIa dose
Not Known	NCT03958656	SLAMF7	I	RR/MM ( ≥3 prior regimens)	Safety by frequency of AEs
ATLCAR. CD138 cell	NCT03672318	CD138	I	RR/MM (up to 2 treatment lines if refractory to both IMiD and PI)	Proportion of participants with DLTs as a measure of MTD

AE: adverse event; ASCT: autologous stem cell transplant; CAR: chimeric antigen receptor; DLT: dose-limiting toxicity; IMiD: immunomodulatory agent; MTD: maximum tolerated dose; R/R: relapsed/refractory; PI: proteasome inhibitor.

**Table 5:** CAR T-cell therapy in MM: open questions.

Issue	
CAR T-cell therapy failure	Reasons for failure: Malignancy; Immune-associated; Patient-factors; Antigen escape. Strategies to overcome antigenic loss: Sequential or combined infusion with CAR T-cells against targets other than BCMA; CAR T-cells with novel dual-targeting vector design; BCMA expression upregulation; New potential targets of immunotherapy: CD138, GPRC5D, transmembrane activator, calcium-modulator, cyclophilin ligand, signaling lymphocytic activation, and molecule); NKG2D ligands, CD229 and integrin β; Moving CAR T-cell therapy into the earlier treatment lines.
Safety	CRS: tended to be grade 1 or 2; NT has not been a significant issue in MM CAR T-cell trials, Cytopenias; Hypogammaglobulinemias; The possibility that CAR T therapies are managed on an outpatient basis or with a reduction in the days of hospitalization.
Extended timeline of the manufacturing process	Solutions: Novel bioengineering methods; Lymphocytes from allogeneic donors; Use of induced pluripotent stem cell (iPSC)-derived immune cells; CAR NK cells against.
Access to therapy	Accredited centers are required; ICU and Neurology services; Support social; High cost.
Patient selection	Patients with a lower disease burden to prevent early relapse.

## Data Availability

Data sharing not applicable. No new data were created or analyzed in this study.
